# Explainable Tensor Multi-Task Ensemble Learning Based on Brain Structure Variation for Alzheimer’s Disease Dynamic Prediction

**DOI:** 10.1109/JTEHM.2022.3219775

**Published:** 2022-11-04

**Authors:** Yu Zhang, Tong Liu, Vitaveska Lanfranchi, Po Yang

**Affiliations:** Department of Computer ScienceThe University of Sheffield 7315 Sheffield S10 2TN U.K.

**Keywords:** Alzheimer’s disease, multi-task learning, brain biomarker spatio-temporal correlation, tensor decomposition, gradient boosting ensemble learning

## Abstract

Machine learning approaches for predicting Alzheimer’s disease (AD) progression can substantially assist researchers and clinicians in developing effective AD preventive and treatment strategies. This study proposes a novel machine learning algorithm to predict the AD progression utilising a multi-task ensemble learning approach. Specifically, we present a novel tensor multi-task learning (MTL) algorithm based on similarity measurement of spatio-temporal variability of brain biomarkers to model AD progression. In this model, the prediction of each patient sample in the tensor is set as one task, where all tasks share a set of latent factors obtained through tensor decomposition. Furthermore, as subjects have continuous records of brain biomarker testing, the model is extended to ensemble the subjects’ temporally continuous prediction results utilising a gradient boosting kernel to find more accurate predictions. We have conducted extensive experiments utilising data from the Alzheimer’s Disease Neuroimaging Initiative (ADNI) to evaluate the performance of the proposed algorithm and model. Results demonstrate that the proposed model have superior accuracy and stability in predicting AD progression compared to benchmarks and state-of-the-art multi-task regression methods in terms of the Mini Mental State Examination (MMSE) questionnaire and The Alzheimer’s Disease Assessment Scale-Cognitive Subscale (ADAS-Cog) cognitive scores. Brain biomarker correlation information can be utilised to identify variations in individual brain structures and the model can be utilised to effectively predict the progression of AD with magnetic resonance imaging (MRI) data and cognitive scores of AD patients at different stages.

## I. Introduction

Alzheimers’s disease (AD) is a severe primary neurodegenerative disease in which neurons and their connections deteriorate over time, leading to a full spectrum of dementia including cognitive decline, memory loss and executive dysfunction [1]. There is currently no cure to treat or reverse the progression of the disease and it puts patients and their families under enormous psychological and emotional stress. Numerous studies have been conducted to recognize sensitive and precise biomarkers of early Alzheimer’s disease progression that will assistance in early AD diagnosis to create, evaluate and validate current and new treatments.

Utilising machine learning methods to predict AD progression can greatly support clinicians and researchers in making effective disease prevention and treatment decisions. Standard AD prediction methods rely on quantifying and extracting important biomarkers from diverse modalities (e.g., Magnetic Resonance Imaging (MRI) and Positron Emission Tomography (PET)), and then learning the model as a regression problem to calculate cognitive scores at different time points. Existing AD progression models mainly utilise machine learning regression algorithms [2], [3], statistical probability-based survival models [4], [5] and deep learning methods based on neural networks [6], [7]. The input features of the above models are signified as second-order matrices containing patient and biomarker information, and this data representation makes it difficult to predict and analyse disease progress from multiple dimensions (e.g., spatial and temporal dimensions). At the same time, the second-order matrix can only focus on a single biomarker, which will lose the correlation information between different AD biomarkers.

**FIGURE 1.**
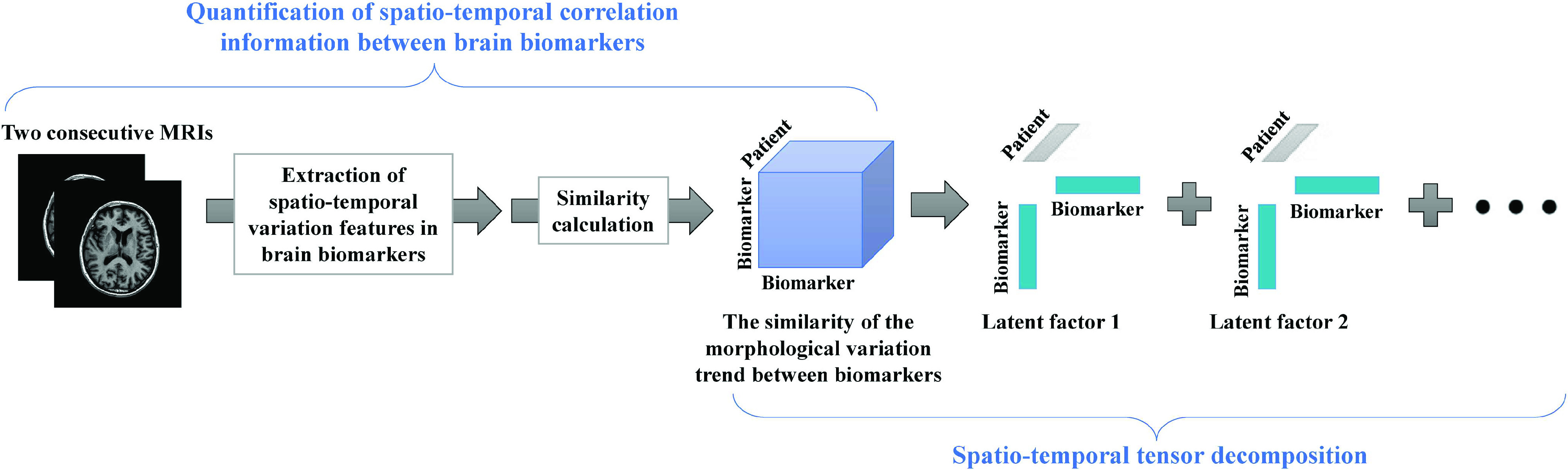
CP decomposition on a spatio-temporal tensor representation based on the similarity of the morphological variation trend between brain biomarkers.

Therefore, instead of applying a second-order matrix with two components for each index, we constructed a third-order tensor with three components for each index (Fig. 1). The paper proposes to build a third-order tensor to build an AD prediction model to better present numerous aspects of AD data in both spatial and temporal dimensions. With the enhanced presentation of AD biomarker features, the utilise of the tensor in regression algorithms can improve prediction accuracy, stability and interpretability. Secondly, for AD prediction models, multi-task learning (MTL) can share information across tasks, outperforms traditional single-task learning methods in terms of prediction accuracy, interpretability and generalisation, and is most effective when the number of samples is small [8]. Therefore, we designed a tensor-based MTL approach to predict AD progression by incorporating spatio-temporal information on brain structural variations. Specifically, we first propose a method for quantifying structural variations in the brain based on similarity calculations, which expresses the similarity of morphological variation trends between biomarkers as a third-order tensor with dimensions corresponding to the first biomarker, the second biomarker and the patient sample. Subsequently, the proposed algorithm performs a CANDECOMP/PARAFAC (CP) decomposition of the tensor [9] and extracts a set of rank-one latent factors from the data. As shown in Fig. 1, the similarity in morphological variation trends between biomarkers can be decomposed into a set of rank-one tensors, each calculated from the outer product of three rank-one latent factors. Each latent factor is described by its first biomarker, second biomarker and patient sample dimension, resulting in an interpretable way to describe the latent factors controlling the variability of the data, and the latent factors can be utilised as predictors for training the MTL model.

In addition to the above challenges, in real-life applications, patients with suspected AD will continue to go to hospital for testing, which is a waste of subsequent incremental data if only a baseline model is utilised or if continuous testing records of the patient cannot be reasonably integrated. To address this problem, we utilise a gradient boosting ensemble learning approach to integrate consecutive test records of subjects to further improve prediction accuracy.

The contributions of this article are summarized as follows:

1) A similarity measurement method for quantifying and understanding variability in AD brain structure data is proposed to extract temporal and spatial information between biomarkers, further combined with tensor decomposition to obtain latent factors.
2) The proposed tensor-based MTL algorithm seamlessly integrates spatio-temporal information based on brain structural variations and its biomarker latent factors, thus significantly improving the predictive accuracy and stability of AD progression.
3) Identified and analysed important spatio-temporal variation correlations between brain biomarkers in the AD progression.
4) The proposed AD dynamic prediction utilises gradient boosting ensemble learning to combine multiple consecutive MRI detections and the experimental results demonstrate that the prediction accuracy continues to improve as the number of MRI detections increases.

## II. Related Work

Numerous preceding brain science studies have focused on the differences in brain structure variation of AD, CN (cognitively normal older individuals) and MCI (mild cognitive impairment). [10] developed a distortion-based framework for modelling the properties of AD and aging on the morphological progression of the brain, emphasizing specific morphological changes in the brain to help identify clinical conditions. [11] evaluated the correlation of CSF and MRI biomarkers with clinical diagnosis and cognitive functioning in patients with CN, AD and aMCI (amnestic mild cognitive impairment). It was concluded that MRI provided stronger cross-sectional grouping, discrimination and correlated better with cross-sectional integrated cognitive and functional abilities. [12] used automated MRI analysis to evaluate cortical thickness in healthy older adults, MCI patients and AD patients. Patterns of cortical thinning were identified as a function of disease progression, and it was discovered that as the disease marched from MCI to AD, the whole cortex thinned and extended appreciably into the lateral temporal cortex.

In addition, the research on correlations between AD MRI biomarkers has been a focus of brain structural variation research, [13] used correlation of multi-kernel support vector machines and regional mean cortical thickness to combine relevant information with ROI-based data to advance the classification performance of AD and its precursor stages. [14] structured brain networks by thresholding the cortical thickness correlation matrix for different regions and analysed them using graph theory. The above study evaluated and analysed the relationship between AD progression and brain biomarkers and showed that there are differences between brain biomarkers for AD, MCI and CN. However, the above studies only focus on a particular biomarker or the same category of biomarker, lacking the linkage and correlation of spatio-temporal variation between dissimilar categories of biomarkers, which is important for AD feature representation.

The AD prediction can be considered as a multi-task regression problem [15]. The primary assumption of the model is that there is an intrinsic link between a large number of data records and that capturing the intrinsic link enhances the generalizability of the predictive model. The sharing of information between different patient prediction tasks promises advance achievable performance. This advantage is particularly pronounced when the number of input features (e.g., AD biomarkers) exceeds the number of samples (e.g., patient samples) [16]. In the field of MTL for AD, existing approaches have focused on modelling relationships between tasks using novel regularisation techniques [17], [18]. Kernel methods were added to the technique to enable it to fit non-linear relationships [19], [20]. The aforementioned study and experiments demonstrated that the regularised MTL technique performs well in a diversity of AD prediction applications.

To the best of our knowledge, there is no commonly exercised tensor regression algorithm that exploits the multidimensional properties of biomarker data to predict AD progression. Current third-order tensor-based algorithms in AD are mainly used for images (e.g., MRI) [21] and electroencephalograms (EEG), as the images themselves are third-order tensors and each patient’s EEG can be composed of a third-order tensor in the time and frequency domains. But in terms of the form of the data, MRI brain biomarker data can be formed into a third-order tensor. The first characteristic of data that can be constructed as a tensor is that it is multidimensional data, and the second characteristic is that the original data is inherently a tensor, for example as an RGB image it is innately a three-dimensional tensor. Brain biomarker data are multidimensional data that fit the first characteristic of tensor data. Based on this fact, we have pioneered a tensor-based MTL method for accurate AD progression prediction.

## III. Methodology

### A. Denotation

For brevity, we represent tensors as italic capital letters, such as $X$ or $Y$, and matrices by capital letters, such as A or B. Vectors are denoted by lowercase letters such as x whereas Scalars are denoted by italic lowercase letters such as $a$.

### B. Spatio-Temporal Variation Similarity Calculation of MRI Biomarkers

Two successive MRI tests were utilised to calculate the spatio-temporal variation in brain biomarkers. The quantitative approach has been reported in its preliminary versions [22], [23], and this research expands and exploits it across number of successive time points (BL to M06, M06 to M12, M12 to M24). For instance, at time points baseline (the date the patient was first screened in hospital) and M06 (the time point six months after the first visit), we utilised MRI at the corresponding time points to calculate the rate of change and velocity for each biomarker, where $x$ is the test value of brain biomarkers and $t$ is the MRI detection dates. The rate of change is $\frac {x_{M06}-x_{BL}}{x_{BL}}$, the velocity is $\frac {x_{M06}-x_{BL}}{t_{M06}-t_{BL}}$ per month. The rate of change and velocity were then utilised to create a vector which describes the morphological variation trend of brain biomarker.

In this study, the similarity between two vectors was calculated utilising the Mahalanobis distance as a method to indicate the similarity of the spatio-temporal variation of two MRI biomarkers. The Mahalanobis distance was utilised because it is scale-independent when the covariance matrix is divided [24]. The Mahalanobis distance between the vectors $\mathrm {x}_{i}$ and $\mathrm {x}_{j}$ is defined as: $\mathrm {Ma}\left ({\mathrm {x}_{i},\mathrm {x}_{j} }\right)=\sqrt {\left ({\mathrm {x}_{i}-\mathrm {x}_{j} }\right)^{\mathrm {T}}\mathrm {S}^{-1}(\mathrm {x}_{i}-\mathrm {x}_{j})}$, where S is covariance matrix.

**FIGURE 2.**
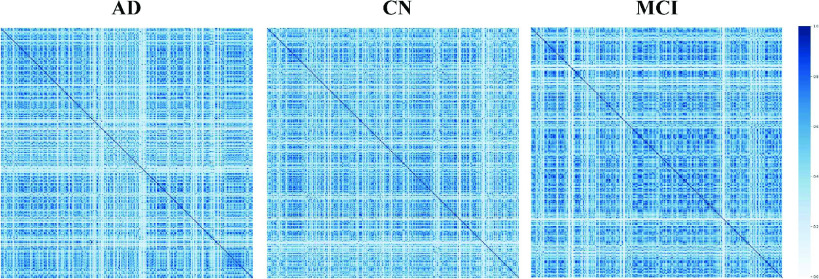
Examples of Mahalanobis distance matrix distribution for AD, CN and MCI brain biomarker relationships. The difference in the matrix areas is evident in the figure, which indicates the difference in spatial changes in the brains of AD, CN and MCI as time progresses. (The scale from top to bottom is 1.0, 0.8, 0.6, 0.4, 0.2, 0.0).

Fig. 2 shows the spatio-temporal correlations of brain biomarkers of AD, CN and MCI calculated by Mahalanobis distances. Although similarity calculations can demonstrate differences in the spatio-temporal correlation of AD, CN and MCI brain biomarkers, there is a unifying problem that half of the data is duplicated due to pairings of biomarker association, it may increase the computational complexity and this study addresses this problem utilising the duplicate data correction matrix in the algorithm design.

### C. Tensor Decomposition

Our proposed formula requires an understanding of the latent factors of the correlation tensor of morphological variation trends between MRI biomarkers. These latent factors are represented by factor matrices A and B, which can be derived utilising tensor decomposition methods. There are two mainstream standard approaches for tensor decomposition, specifically Tucker and CANDECOMP/PARAFAC (CP) decomposition [9]. The Tucker decomposition decomposes the tensor into the result of the core tensor and the factor matrix for each mode. Although it expresses a more inclusive statement, it is problematic to interpret the latent factors, as the amount of latent factors may differ for different model. By comparison, CP decomposition decomposes the tensor into a set of rank-one tensors. i.e., $X\mathrm {\approx }\left [{\left [{ \mathrm {A \times B \times C} }\right]}\right]=\sum \nolimits _{i=1}^{r} {\mathrm {a}_{i}\circ \mathrm {b}_{i}\circ \mathrm {c}_{i}}$, where $\circ $ denote the outer product operation between two vectors, while $\mathrm {a}_{i}\mathrm {, }\mathrm {b}_{i}~\mathrm {and}~\mathrm {c}_{i}$ correspond to the vectors related with the $i$-th latent factor. Given a tensor $X$ of the size $n_{1}\times n_{2}\mathrm {\times }n_{3}$, the size of matrix A, B and C is $n_{1}\times r,n_{2}\mathrm { \times }r\,\,\mathrm {and}\,\,n_{3}\times r$ respectively.

### D. Tensor Multi-Task Regression

To predict cognitive scores (e.g., MMSE and ADAS-Cog) at future time points. Consider a tensor multi-task regression problem with $t$ time points, $n$ training samples with $d_{1}$ and $d_{2}$ features. Let $X\,\,\in \mathrm {\mathbb {R}}^{d_{1}\mathrm {\times }d_{2}\times n}$ be the input tensor from two consecutive MRI tests and the it is the amalgamation of similarity matrix of all $n$ samples $\mathrm {X}_{n}\mathrm {\in }\mathrm {\mathbb {R}}^{d_{1}\times d_{2}}$, $\mathrm {Y=[}\mathrm {y}_{1}\mathrm {,\cdots,}\mathrm {y}_{t}\mathrm {]\in }\mathrm {\mathbb {R}}^{n\times t}$ be the targets and $\mathrm {y}_{t}=[y_{1}\mathrm {,\cdots,}y_{n}\mathrm {]\in }\mathrm {\mathbb {R}}^{n}$ is the corresponding target (clinical scores) at different time points. We utilise the operator ${\odot }$ as follows: ${\mathrm {Z=M}} \odot {\mathrm {N}}$ denotes $z_{ij}=m_{ij}n_{ij}$, for all *i, j.*

The input tensor for the similarity of morphological variation trends in brain biomarkers is a symmetric tensor because the relationships between biomarkers are paired and therefore half of the data are duplicated. The research further proposes a duplicate data correction matrix to resolve the problem of duplicate data and it states as follows:

\begin{align*} \text {K} = \left [{ {\begin{array}{cccccccccccccccccccc} 0 & ~{\begin{array}{cccccccccccccccccccc} 1 &~\cdots \\ \end{array}} &~1\\ \vdots &~\ddots & ~{\begin{array}{l} \vdots \\ 1 \\ \end{array}}\\ 0 &~\cdots &~0\\ \end{array}} }\right]\in \mathbb {R}^{d_{1}\times d_{2}}\tag{1}\end{align*}

For $t$-th prediction time point, the objective function of the proposed approach can be stated as follows:

\begin{align*} L_{t}\left ({X, y_{t}}\right)=&\min _{W_{t}, A_{t}, \mathrm {B}_{t}, \mathrm {c}_{t}} \frac {1}{2}\left \|{\hat {y}_{t}-y_{t}}\right \|_{\mathrm {F}}^{2}+\frac {\lambda }{2} \\[-2pt]&\left \|{X-[\![\mathrm {A}_{t}, \mathrm {~B}_{t}, \mathrm {C}_{t}]\!]}\right \|_{\mathrm {F}}^{2} +\beta \left \|{\mathrm {W}_{t}, \mathrm {~A}_{t}, \mathrm {~B}_{t}, \mathrm {C}_{t}}\right \|_{1} \\[-2pt] \hat {y}_{n}=&\sum \nolimits _{i=1}^{d_{1}} \sum \nolimits _{j=1}^{d_{2}} \mathrm {U}_{i j},\end{align*}

where

\begin{equation*} \mathrm {U}=(\mathrm {A}_{t}\mathrm {B}_{t}^{\mathrm {T}})\odot \mathrm {K\odot }\mathrm {W}_{t}\mathrm {\odot }\mathrm {X}_{n}, \mathrm {U}\in \mathbb {R}^{d_{1}\times \acute{\text{A}}d_{2}}.\tag{2}\end{equation*}

where the first term computes the empirical error for the training data, $\hat {y}_{t}=\left [{ \hat {y}_{1}\mathrm {,\cdots,}\hat {y}_{n} }\right]\in \mathrm {\mathbb {R}}^{n}$ are the predicted values, $\mathrm {A}_{t}\in \mathrm {\mathbb {R}}^{d_{1}\times r}$ is the latent factor matrix for first biomarker dimension and $\mathrm {B}_{t}\in \mathrm {\mathbb {R}}^{d_{2}\times r}$ is the latent factor matrix for second biomarker dimension with r latent factors, $\mathrm {W}_{t}\mathrm {\in }\mathrm {\mathbb {R}}^{d_{1}\times d_{2}}$ is the model parameter matrix for $t$-th prediction time point, $\lambda $ and $\beta $ are the regularization parameters. Obtaining latent factors by optimising objective function $\left \|{ X-\left [{\left [{ \mathrm {A, B, C} }\right]}\right] }\right \|_{\mathrm {F}}^{2}$, where $X=\left [{\left [{ \mathrm {A, B, C} }\right]}\right]=\sum \nolimits _{i=1}^{r} {\mathrm {a}_{i}\circ \mathrm {b}_{i}\circ \mathrm {c}_{i}}$ where $\circ $ denote the outer product operation between two vectors. $\left \|{ \mathrm {W}_{t},\mathrm {A}_{t},\mathrm {B}_{t},\mathrm {C}_{t} }\right \|_{1}$ employing an $l1$-norm on the W_*t*_, A_*t*_, B_*t*_ and C_*t*_ matrices respectively.

For all prediction time points, the objective function can be stated as follows:

\begin{equation*} L(X, \mathrm {Y})=\min _{\mathrm {W}_{f}} \sum \nolimits _{1}^{t} L_{t}\left ({X, \mathrm {y}_{t}}\right)+\theta \left \|{\mathrm {W}_{f} \mathrm {P}(\alpha)}\right \|_{\mathrm {F}}^{2}\tag{3}\end{equation*}

where $\left \|{\mathrm {W}_{f} \mathrm {P}(\alpha)}\right \|_{\mathrm {F}}^{2}$ is the generalized temporal smoothness term, model parameter matrix $\mathrm {W}_{f}\mathrm {\in }\mathrm {\mathbb {R}}^{(d_{1}\times d_{2}\mathrm {)\times }t}$is the temporal dimension unfolding for model parameter tensor $W\,\,\in \mathrm {\mathbb {R}}^{d_{1}\times d_{2}\mathrm {\times }t}$, $\theta $ is the regularization parameter. The generalised temporal smoothing states the fact that in actually diagnosing AD, the specialist not only relies on the patient’s current symptoms, but also takes into account their previous symptoms. Therefore, we assume that the $i$-th progression in an individual AD patient is related to all preceding progressions. The generalized temporal smoothness prior describe as follows:

\begin{align*} {\begin{cases} \Delta w_{1}=\delta w_{1} \\[-2pt] \Delta w_{2}=\alpha _{1}\Delta w_{1}+(1-\alpha _{1})\delta w_{2} \\[-2pt] \Delta w_{3}=\alpha _{2}\Delta w_{2}+(1-\alpha _{2})\delta w_{3} \\[-2pt] \cdots \\[-2pt] \Delta w_{t-1}=\alpha _{t-2}\mathrm {\Delta }w_{t-2}+(1-\alpha _{t-2})\delta w_{t-1} \\[-2pt] \end{cases}}\tag{4}\end{align*}

where $\Delta w$ denoted the progression with preceding progression information. $w_{i}$ is the $i$-th column of W. where the parameter $\alpha $ represents the relational degree of the $i$-th progression and all preceding progressions. In addition, the impact of each stage of disease progression on the following stage may not be consistent, and therefore the relational degree parameters differ for each disease progression stage. The definition of the $i$-th progression $\delta w_{i}$ for one patient is:

\begin{equation*} \delta w_{i}=w_{i}-w_{i+1},i=1,2,\cdots,t-1\tag{5}\end{equation*}

As a result, we can describe the more realistic temporal smoothness assumption with matrix multiplication:

\begin{equation*} \text {WP}(\alpha \mathrm {)=WH}\mathrm {D}_{1}(\alpha _{1})\mathrm {D}_{2}(\alpha _{2}\mathrm {)\cdots }\mathrm {D}_{t-2}(\alpha _{t-2})\tag{6}\end{equation*}

where $\mathrm {H\in }\mathrm {\mathbb {R}}^{t\times \acute{\text{A}}(t\mathrm {-1)}}$ has the following definition: ${\mathrm {H}_{ij}=1 \,\,\text {if} } i=j,\mathrm {H}_{i}=-1$ if $i=j+1$ and $\mathrm {H}_{ij}\mathrm {=0}$ otherwise. $\mathrm {P(}\alpha)$ denotes the correlation between progress, it comprises the hyperparameters $\alpha $, which depends on the result of cross-validation. $\mathrm {D}_{i}(\alpha _{i}\mathrm {)\in }\mathrm {\mathbb {R}}^{(t-1)\times \acute{\text{A}}(t\mathrm {-1)}}$ is an identity matrix and the value of $\mathrm {D}_{i_{m,n}}(\alpha _{i})$ is substituted by $\alpha _{i}$ if $m=i,n=i+1$, the value of $\mathrm {D}_{i_{m,n}}(\alpha _{i})$ is substituted by $1-\alpha _{i}$ if $m=n=i+1$.

Latent factors $A \in \mathrm {\mathbb {R}}^{d_{1}\mathrm {\times }r\times t}$, $B \in \mathrm {\mathbb {R}}^{d_{2}\mathrm {\times }r\times t}$, $C \in \mathrm {\mathbb {R}}^{d_{3}\mathrm {\times }r\times t}$ and the model parameter $W\mathrm {\in }\mathrm {R}^{d_{1}\times d_{2}\times t}$ can be learned by iteratively optimising the objective function for each set of variables to be solved. Because not all components of the objective function are differentiable, we utilise proximal gradient descent to solve each subproblem. Specifically, the terms in our objective function involving Frobenius norms are differentiable, but those involving the sparsity-inducing $l1$-norms are not differentiable. In the MTL model, the proximal approach is frequently utilised to construct the proximal issue for the non-smooth objective function [25], [26], [27], [28], by replacing the smooth function with the quadratic function, we get the sum of the smooth and non-smooth functions. Its quadratic functions can be constructed in a variety of ways based on Taylor series, and the resulting proximal problems are usually easier to solve than the original ones. The strategy can simplify the design of distributed optimisation algorithms or accelerate the convergence of the optimisation process.

### E. Gradient Boosting

Ensemble learning has been proven to be effective in a variety of prediction tasks by grouping a set of weak learners together to build stronger learners. Boosting is the dominant technique in ensemble learning methods, which produces a set of weak learners in which predictors are trained sequentially rather than individually, with the aim of utilising the errors of the previous learner to develop a more effective model for the next learner.

**FIGURE 3.**
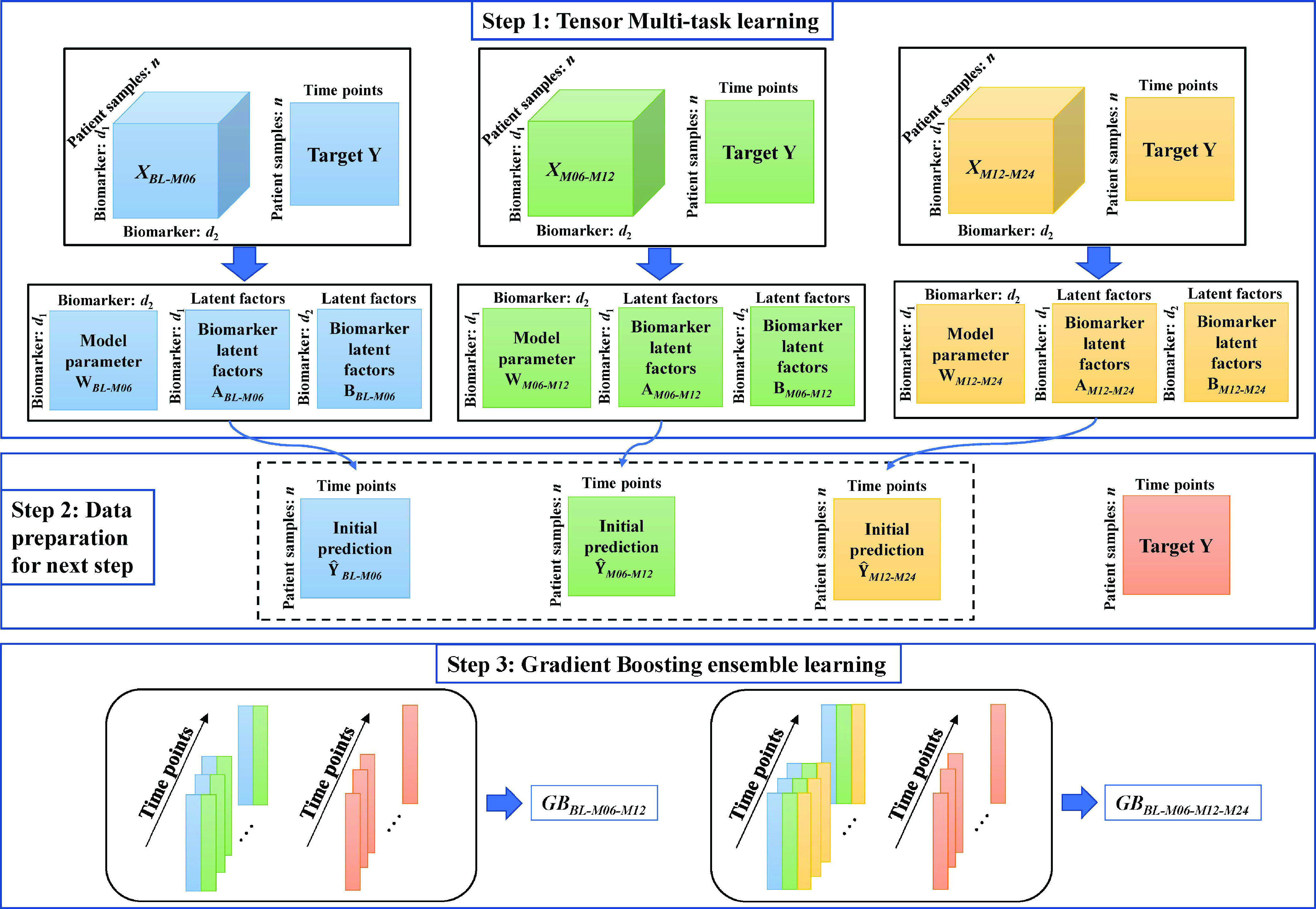
The flowchart of the proposed method in the training stage.

Gradient Boosting (GB) is an extension of the boosting method which utilises gradient descent optimisation techniques to identify global or local minima of the cost function. It trains the machine to fit the model on the input feature space through a series of weak learners, each of which improves the prediction accuracy of the previous learner. GB trains powerful learners by combining numerous weak learners in multiple iterations [29], [30]. The proposed method enhances prediction accuracy by sequentially fitting a more accurate model to the residuals of the preceding step in the final stage of the GB construction framework. This process will continue until a highly accurate model is obtained. The flowchart of the proposed method in the training stage is illustrated in Fig. 3.

## IV. Experimental Settings

### A. Dataset

Data used in the preparation of this article were obtained from the Alzheimer s Disease Neuroimaging Initiative (ADNI) database (adni.loni.usc.edu). The ADNI was launched in 2003 as a public-private partnership, led by Principal Investigator Michael W. Weiner, MD. The primary goal of ADNI has been to test whether serial magnetic resonance imaging (MRI), positron emission tomography (PET), other biological markers, and clinical and neuropsychological assessment can be combined to measure the progression of mild cognitive impairment (MCI) and early Alzheimer s disease (AD). The FreeSurfer image analysis software (https://surfer.nmr.mgh.harvard.edu/) was used by a team from the University of California, San Francisco (UCSF) to conduct volumetric segmentations and cortical reconstruction using imaging data from the ADNI database, which includes all ADNI subprojects (ADNI 1, 2, GO, 3). We gained the MRI data from the ADNI website and continued to implement the subsequent pre-processing steps:

• Removal of features with missing values in more than half of the sample;
• Exclusion of individual participants who did not have BL and M06 MRI;
• Missing data were filled with average of the features;
• Removal of cognitive function assessments for individuals with missing follow-up points in longitudinal studies;
• For AD dynamic prediction, exclude individuals who did not have follow-up MRI detections.

After the pre-processing steps, there are a total of 313 MRI features. which can be classified into five categories: the volumes of cortical parcellations (CV), the volumes of specific white matter parcellations (SV), the total surface area of the cortex (SA), average cortical thickness (TA) and standard deviation in cortical thickness (TS). Table 1 depicts the demographic features of the ADNI MRI data utilised in this research.

**TABLE 1:** Demographic Characteristic of the Studied Subjects Valued are Specified as Mean ± Standard Deviation. (The Notation “M12” Indicates the Time Point 12 Months After the First Visit, “M24” Indicates the Time Point 24 Months After the First Visit, etc)

Time point	Attribute	MMSE	ADAS-Cog
M12	Sample size (CN, MCI, AD)	1332 (358, 725,249)	1299 (351, 716,232)
	Gender(f/m)	579/753	565/734
	Age	75.0±7.1	75.1±7.1
M24	Sample size (CN, MCI, AD)	1110(330, 617,163)	1066 (321, 602,143)
	Gender(f/m)	484/626	464/602
	Age	76.0±7.2	73.±7.1
M36	Sample size (CN, MCI, AD)	710 (192,509, 9)	677 (186,485, 6)
	Gender(f/m)	307/403	292/385
	Age	76.7±7.0	73.5±7.0
M48	Sample size (CN, MCI, AD)	456 (120, 334, 2)	431 (114,315, 2)
	Gender(f/m)	195/261	185/246
	Age	77.1±6.9	73.1±6.9

For the predictive target of the approach, cognitive scores (MMSE and ADAS-Cog) can be used to precisely differentiate between CN, MCI and AD in the clinical scenarios. For MMSE with score range 0–30, 30 is represented as cognitively no dementia, 29–26 is represented as questionable dementia, 25–21 is represented as mild dementia, 20–11 is represented as moderate dementia, 10–0 is represented as severe dementia. For ADAS-Cog with score range 0–70, where higher scores indicate greater cognitive impairment.

### B. Evaluation Metrics

The similarity tensor of morphological variation trends between MRI brain biomarkers was utilised to build predictive models for each target. The data was randomly divided into a training set and a test set in a ratio of 9:1. As the number of model parameters ( $\lambda $, $\beta $ and $\theta$), the hyperparameters $\alpha $ and the latent factor $r$ must be designated during the training phase, we utilise 5-fold cross-validation on the training data to select them. The research evaluates the prediction performance of various methods for each single time point utilising the root mean square error (rMSE) as the major evaluation metric. We use normalised mean square error (nMSE) for the overall regression performance metrics, which is commonly utilised in multi-task learning research [31]. The rMSE and nMSE are stated as follows:

\begin{align*} \mathrm {rMSE}(\mathrm {y}, \hat {y})=&\sqrt {\frac {\| \mathrm {y}-\hat {\mathrm {y}}_{2}^{2}}{n}} \tag{7}\\ \mathrm {nMSE}(\mathrm {Y}, \hat {\mathrm {Y}})=&\frac {\sum _{i=1}^{t}\left \|{\mathrm {Y}_{i}-, \widehat {\mathrm {Y}}_{i}}\right \|_{2}^{2} / \sigma \left ({\mathrm {Y}_{i}}\right)}{\sum _{i=1}^{t} n_{i}}\tag{8}\end{align*}

where for rMSE, y is the ground truth of target at a single time point and $\hat {y}$ is the corresponding prediction by a model. For nMSE, Y_*i*_ is the target’s ground truth at time point $i$ and $\hat {Y}_{i}$ is the corresponding prediction from a model. We reported the mean and standard deviation based on 20 iterations of experiments on dissimilar splits of data.

## V. Results and Discussion

### A. Comparison With the Benchmarks and State-of-the-Arts

We utilised the Mahalanobis distance to construct a tensor of morphological variations in the brain, combined with the proposed tensor multi-task ensemble learning (TMTL-GB) algorithm to compare with single task learning, benchmarks and state-of-the-art multi-task learning algorithms that were chosen as competitive methods in studies to predict clinical deterioration, including Ridge regression (Ridge) [32], Lasso regression (Lasso) [33], Temporal Group Lasso (TGL) [8], Non-convex Fused Sparse Group Lasso (nFSGL1) [34], Convex Fused Sparse Group Lasso (cFSGL) [8], Non-Convex Calibrated Multi-Task Learning (NC-CMTL) [35], Fused Laplacian Sparse Group Lasso (FL-SGL) [36], Joint feature and task aware multi-task feature learning (FTS-MTFL) [37], Group Asymmetric Multi-Task Learning (GAMTL) [38] and Dual feature correlation guided multi-task feature learning (dMTLc) [39]. The experimental results of MMSE and ADAS-Cog predictions are shown in Table 2 and 3.

**TABLE 2:** Comparison of the Results From Our Proposed Methods With Benchmarks and State-of-the-Art Methods for MMSE at Time Points M12 to M48. The Best Results are Bolded

Target: MMSE	Input MRI data	nMSE	M12 rMSE	M24 rMSE	M36 rMSE	M48 rMSE
Ridge	BL, M06	3.1052±0.5803	4.5980±0.5196	5.0259±0.4925	5.5803±0.4948	6.3998±0.7111
	BL, M06, M12	7.5077±1.8073	–	7.8248±1.0884	8.3919±1.1916	8.6505±0.7495
	BL, M06, M12, M24	10.1467±4.9847	–	–	12.5386=1=1.4103	12.0900±1.9673
Lasso	BL, M06	1.0635±0.1408	2.4041±0.2048	2.8315±0.2728	3.3494±0.4011	4.6762±0.5320
	BL, M06, M12	1.1887±0.3307	–	3.0930±0.6897	3.5850±0.8825	4.2383±0.6208
	BL, M06, M12, M24	1.1931±0.4177	–	–	3.5953±0.7182	4.1443±0.9627
TGL	BL, M06	0.7557±0.0948	2.3491±0.2496	2.3924±0.1822	2.6532±0.4482	3.1915±0.2351
	BL, M06, M12	0.6448±0.1526	–	2.2461±0.1647	2.4579±0.2869	2.7096±0.4303
	BL, M06, M12, M24	1.6408±0.2564	–	–	3.9806±0.5370	5.1047±0.4786
nCFGLl	BL, M06	0.4609±0.0694	1.5990±0.2039	1.9405±0.2753	2.2243±0.4086	3.1998±0.4022
	BL, M06, M12	0.3970±0.0257	–	1.5791±0.2059	1.7253±0.1967	2.3118±0.5327
	BL, M06, M12, M24	0.4456±0.1265	–	–	2.0062±0.3586	2.6751±0.5949
cFSGL	BL, M06	0.6899±0.1378	1.9939±0.2025	2.2874±0.2635	2.4874±0.2099	3.1284±0.4442
	BL, M06, M12	0.5472±0.0330	–	1.8646±0.2345	2.0759±0.3413	2.9894±0.3671
	BL, M06, M12, M24	1.1483±0.4289	–	–	3.8737±0.7312	4.1492±1.0407
NC-CMTL	BL, M06	0.4923±0.0990	1.5901±0.2152	1.7454±0.2432	1.9506±0.2389	2.8301±0.4291
	BL, M06, M12	0.5309±0.0547	–	1.8492±0.3216	2.1096±0.3341	3.1799±0.3126
	BL, M06, M12, M24	0.5095±0.0916	–	–	2.1880±0.4971	3.3899±0.2483
FL-SGL	BL, M06	0.6186±0.1324	1.5949±0.2362	1.8812±0.2813	2.0049±0.2533	2.9717±0.5587
	BL, M06, M12	0.5658±0.1009	–	1.8750±0.2796	2.1844±0.2363	3.5419±0.2596
	BL, M06, M12, M24	0.5135±0.0647	–	–	2.4659±0.2989	3.4615±0.5029
FTS-MTFL	BL, M06	0.4434±0.0477	1.6019±0.2208	1.8825±0.2288	2.1584±0.3356	2.7485±0.2815
	BL, M06, M12	0.5166±0.1385	–	1.7910±0.2323	2.1124±0.3644	2.9670±0.6952
	BL, M06, M12, M24	0.4725±0.0982	–	–	2.0178±0.2423	3.1199±0.4141
GAMTL	BL, M06	0.4212±0.0224	1.6861±0.1608	1.7414±0.1827	2.1726±0.3309	3.1652±0.4937
	BL, M06, M12	0.4837±0.0378	–	1.9608±0.2191	2.0752±0.3493	3.0218±0.6099
	BL, M06, M12, M24	0.4327±0.1646	–	–	1.9180±0.1201	2.9964±0.5195
dMTLc	BL, M06	0.4211 ±0.0670	1.4728±0.1852	1.7208±0.0741	2.0877±0.4341	2.7384±0.4462
	BL, M06, M12	0.4459±0.0502	–	1.7908±0.1423	2.1016±0.3219	3.1987±0.2949
	BL, M06, M12, M24	0.4179±0.0387	–	–	2.2954±0.3773	2.7465±0.6070
TMTL-GB	BL, M06	0.3119±0.0421	1.4577±0.1665	1.4959±0.1587	1.5520±0.1450	2.2129±0.2111
	BL, M06, M12	0.3014±0.0574	–	1.2534±0.0561	1.3919±0.0809	2.0594±0.1549
	BL, M06, M12, M24	0.2552±0.0283	–	–	1.2963±0.0584	1.9356±0.0146

**TABLE 3:** Comparison of the Results From Our Proposed Methods With Benchmarks and State-of-the-Art Methods for ADAS-Cog at Time Points M12 to M48. The Best Results are Bolded

Target: ADAS-Cog	Input MRI data	nMSE	M12 rMSE	M24 rMSE	M36 rMSE	M48 rMSE
Ridge	BL, M06	1.3773±0.1298	9.4358±0.9725	9.7975±1.1718	10.9876±1.3781	14.6598±1.8363
	BL, M06, M12	1.5331±0.2192	–	10.4089±1.4291	11.5586±1.5590	15.1463±1.9747
	BL, M06, M12, M24	1.7338±0.1754	–	–	12.1387±1.5305	14.8322±1.3309
Lasso	BL, M06	0.7974±0.0917	6.4992±0.5238	6.9227±1.1271	8.6765±1.0948	11.9426±1.6025
	BL, M06, M12	0.7631±0.0937	–	6.6594±0.5650	8.9510±1.2444	11.3838±1.4890
	BL, M06, M12, M24	0.7186±0.0912	–	–	8.4415±1.2726	10.4232±1.6738
TGL	BL, M06	0.3688±0.0424	4.7842±0.3764	4.6610±0.5169	5.7494±0.5775	7.7067±1.2795
	BL, M06, M12	0.4229±0.0806	–	5.4470±0.5740	5.5116±0.7395	8.5023±1.8449
	BL, M06, M12, M24	0.2772±0.0315	–	–	4.4827±0.3285	7.1983±1.1176
nCFGLl	BL, M06	0.2255±0.0256	3.6844±0.2590	3.4506±0.2410	3.8452±0.1968	7.5590±1.5356
	BL, M06, M12	0.2144±0.0462	–	3.8132±0.7284	3.6613±0.2673	6.4116±0.8013
	BL, M06, M12, M24	0.2327±0.0443	–	–	3.9810L0.4929	6.5759±1.9124
cFSGL	BL, M06	0.3623±0.0488	4.1972±0.2893	5.0202±0.5373	5.3536±0.5622	8.2545±2.1690
	BL, M06, M12	0.2801±0.0532	–	4.1486±0.4266	5.0682±0.3117	6.3823±1.0508
	BL, M06, M12, M24	0.2359±0.0343	–	–	4.3043±0.5869	6.9518±1.6547
NC-CMTL	BL, M06	0.2386±0.0351	3.7329±0.4119	4.0644±0.6422	4.2489±0.4014	5.6502±0.9988
	BL, M06, M12	0.1757±0.0226	–	3.3772±0.2310	4.0025±0.3989	4.9068±0.2093
	BL, M06, M12, M24	0.1955±0.0217	–	–	3.9598±0.2237	5.9555±1.1476
FL-SGL	BL, M06	0.2505±0.0423	4.4357±0.6086	4.1841±0.3426	4.7807±0.5122	6.8256±0.3117
	BL, M06, M12	0.3197±0.0457	–	4.9369±0.4912	4.3522±0.7789	6.8449±0.5871
	BL, M06, M12, M24	0.2666±0.0140	–	–	4.7754±0.3305	8.1430L0.8817
FTS-MTFL	BL, M06	0.2360±0.0306	3.9568±0.3706	4.0168±0.5553	4.1329±0.2210	6.4760±1.4213
	BL, M06, M12	0.2051±0.0226	–	3.6510±0.3699	4.3284±0.5781	6.0530±1.4375
	BL, M06, M12, M24	0.2252±0.0539	–	–	3.8780±0.4388	5.6164±1.0738
GAMTL	BL, M06	0.2146±0.0381	3.9110±0.3454	3.1081±0.5278	3.7255±0.4643	6.4080±0.9305
	BL, M06, M12	0.1963±0.0089	–	3.7110±0.4135	4.3212±0.9916	5.8161±0.9832
	BL, M06, M12, M24	0.2066±0.0341	–	–	4.5519±0.3304	6.5930±0.9055
dMTLc	BL, M06	0.2139±0.0286	4.0192±0.1445	3.4101±0.4066	3.8021±0.5264	6.6308±1.1940
	BL, M06, M12	0.1959±0.0270	–	3.6894±0.2469	3.7553±0.8032	6.7850±0.5438
	BL, M06, M12, M24	0.2007±0.0408	–	–	4.1612±0.6067	6.4822±0.8864
TMTL-GB	BL, M06	0.1919±0.0160	3.4351±03292	3.2926±0.1513	3.6045±0.2307	4.91 ll±0.3109
	BL, M06, M12	0.1673±0.0152	–	3.2294±0.1870	3.6290±0.5421	4.5516±0.6506
	BL, M06, M12, M24	0.1584±0.0254	–	–	3.5645±0.3680	4.2781±0.5841

For overall regression performance, our proposed approach outperforms benchmarks and state-of-the-art approaches in terms of nMSE for both cognitive scores MMSE and ADAS-Cog. And for all individual time points, the proposed approach obtains a smaller rMSE than other approaches. The followings are our main observations: 1) The proposed tensor MTL model outperforms single-task learning models, benchmarks and state-of-the-art MTL models, demonstrating the utilise of morphological variation trend similarity calculations and tensor latent factor hypothesis in our MTL formulation. 2) The proposed tensor MTL method significantly improves the prediction stability. The results obtained through 20 iterations have a lower standard deviation compared to other comparative methods. This may be due to the addition of biomarker latent factors to the prediction algorithm to increase stability. 3) The proposed tensor multi-task ensemble learning can effectively aggregate the temporally continuous MRI records of subjects to improve the prediction accuracy, and with the increase of temporally continuous MRI records, the prediction accuracy increases for subsequent time points. In contrast, the addition of temporally continuous MRI records had no significant effect on benchmarks and state-of-the-art competitive methods.

### B. Interpretability of Spatio-Temporal Relationships Between Biomarkers

Currently, there is no cure for AD, therefore the key to current treatment is early detection and prevention of AD, therefore, identifying important spatio-temporal biomarker relationships in early MRI data can help clinicians to identify patients with suspected AD for early prevention. Tables 4, 5, 6 and 7 provide the top 10 brain biomarker relationships in descending order of weighted parameter values for MMSE prediction (since the MMSE sample size was higher than the ADAS-Cog at all time points) at different time points for the proposed TMTL-GB model. Higher values indicate a greater impact on the final prediction.

**TABLE 4:** The Top-10 Rank Brain Biomarker Relationships With Time Point M12 for the Proposed TMTL-GB Model on MMSE Prediction

Rank	Biomarker relationship	Weight parameter value
1	Vol(C). of R.RostralMiddleFrontal (RMF.R) - Surf. Area of R.InferiorTemporal (ITG.R)	0.4560
2	CTA. of L.SuperiorParietal (SPG.L) - Vol(C). of R.InferiorParietal (IPL.R)	0.4452
3	CTA. of R.ParsTriangularis (PTRI.R) - CTA. of L.Bankssts (BAN.L)	0.4434
4	Vol(WM). of CorpusCallosumMidAnterior (CCMA) - CTA. of L.TransverseTemporal (TTEM.L)	0.4402
5	Vol(WM). of L.Caudate (CAU.L) - CTStd. of R.InferiorTemporal (ITG.R)	0.4274
6	Vol(C). of L.SuperiorFrontal (SFGdor.L) - Surf. Area of L.SuperiorTemporal (STG.L)	0.4154
7	Surf. Area of R.Pericalcarine (CAL.R) - Surf. Area of R.LateralOrbitofrontal (LORB.R)	0.4076
8	Surf. Area of R.Postcentral (PoCG.R) - CTStd. of R.Insula (INS.R)	0.4057
9	Vol(C). of L.ParsTriangularis (PTRI.L) - Vol(WM). of CorpusCallosumMidAnterior (CCMA)	0.4039
10	Surf. Area of R.Paracentral (PCL.R) - Surf. Area of L.Precuneus (PCUN.L)	0.4010

**TABLE 5:** The Top-10 Rank Brain Biomarker Relationships With Time Point M24 for the Proposed TMTL-GB Model on MMSE Prediction

Rank	Biomarker relationship	Weight parameter value
1	CTA. of R.ParsTriangularis (PTRI.R) - CTA. of L.Bankssts (BAN.L)	0.4707
2	Vol(C). of R.RostralMiddleFrontal (RMF.R) - Surf. Area of R.InferiorTemporal (ITG.R)	0.4698
3	Vol(C). of L.SuperiorFrontal (SFGdor.L) - Surf. Area of L.SuperiorTemporal (STG.L)	0.4671
4	Vol(WM). of L.Caudate (CAU.L) - CTStd. of R.InferiorTemporal (ITG.R)	0.4576
5	Vol(WM). of CorpusCallosumMidAnterior (CCMA) - CTA. of L.TransverseTemporal (TTEM.L)	0.4492
6	Surf. Area of R.Paracentral (PCL.R) - Surf. Area of L.Precuneus (PCUN.L)	0.4482
7	CTA. of L.SuperiorParietal (SPG.L) - Vol(C). of R.InferiorParietal (IPL.R)	0.4380
8	Surf. Area of R.Postcentral (PoCG.R) - CTStd. of R.Insula (INS.R)	0.4312
9	Vol(WM). of CorpusCallosumMidAnterior (CCMA) - Surf. Area of R.MiddleTemporal (MTG.R)	0.4196
10	Surf. Area of L.CaudalMiddleFrontal (CMF.L) - CTA. of R.Bankssts (BAN.R)	0.4192

**TABLE 6:** The Top-10 Rank Brain Biomarker Relationships With Time Point M36 for the Proposed TMTL-GB Model on MMSE Prediction

Rank	Biomarker relationship	Weight parameter value
1	Vol(C). of R.RostralMiddleFrontal (RMF.R) - Surf. Area of R.InferiorTemporal (ITG.R)	0.5619
2	CTA. of L.SuperiorParietal (SPG.L) - Vol(C). of R.InferiorParietal (IPL.R)	0.5093
3	CTA. of R.ParsTriangularis (PTRI.R) - CTA. of L.Bankssts (BAN.L)	0.5080
4	Vol(WM). of L.Caudate (CAU.L) - CTStd. of R.InferiorTemporal (ITG.R)	0.5049
5	Vol(C). of L.SuperiorFrontal (SFGdor.L) - Surf. Area of L.SuperiorTemporal (STG.L)	0.4962
6	Vol(WM). of CorpusCallosumMidAnterior (CCMA) - CTA. of L.TransverseTemporal (TTEM.L)	0.4851
7	CTA. of R.ParsTriangularis (PTRI.R) - Surf. Area of R.Lingual (LING.R)	0.4739
8	Surf. Area of R.Paracentral (PCL.R) - Surf. Area of L.Precuneus (PCUN.L)	0.4697
9	Surf. Area of R.Postcentral (PoCG.R) - CTStd. of R.Insula (INS.R)	0.4660
10	Vol(WM). of CorpusCallosumMidAnterior (CCMA) - Surf. Area of R.MiddleTemporal (MTG.R)	0.4659

**TABLE 7:** The Top-10 Rank Brain Biomarker Relationships With Time Point M48 for the Proposed TMTL-GB Model on MMSE Prediction

Rank	Biomarker relationship	Weight parameter value
1	Vol(C). of R.RostralMiddleFrontal (RMF.R) - Surf. Area of R.InferiorTemporal (ITG.R)	0.6197
2	CTA. of R.ParsTriangularis (PTRI.R) - CTA. of L.Bankssts (BAN.L)	0.5943
3	Vol(WM). of L.Caudate (CAU.L) - CTStd. of R.InferiorTemporal (ITG.R)	0.5679
4	CTA. of R.ParsTriangularis (PTRI.R) - Surf. Area of R.Lingual (LING.R)	0.5444
5	Vol(C). of L.SuperiorFrontal (SFGdor.L) - Surf. Area of L.SuperiorTemporal (STG.L)	0.5428
6	Vol(WM). of CorpusCallosumMidAnterior (CCMA) - CTA. of L.TransverseTemporal (TTEM.L)	0.5420
7	CTA. of R.ParsTriangularis (PTRI.R) - Vol(C). of R.LateralOccipital (LOCC.R)	0.5396
8	CTA. of R.ParsTriangularis (PTRI.R) - Vol(WM). of R.Putamen (PUT.R)	0.5131
9	CTA. of L.SuperiorParietal (SPG.L) - Vol(C). of R.InferiorParietal (IPL.R)	0.5111
10	Surf. Area of R.Postcentral (PoCG.R) - CTStd. of R.Insula (INS.R)	0.5106

**FIGURE 4.**
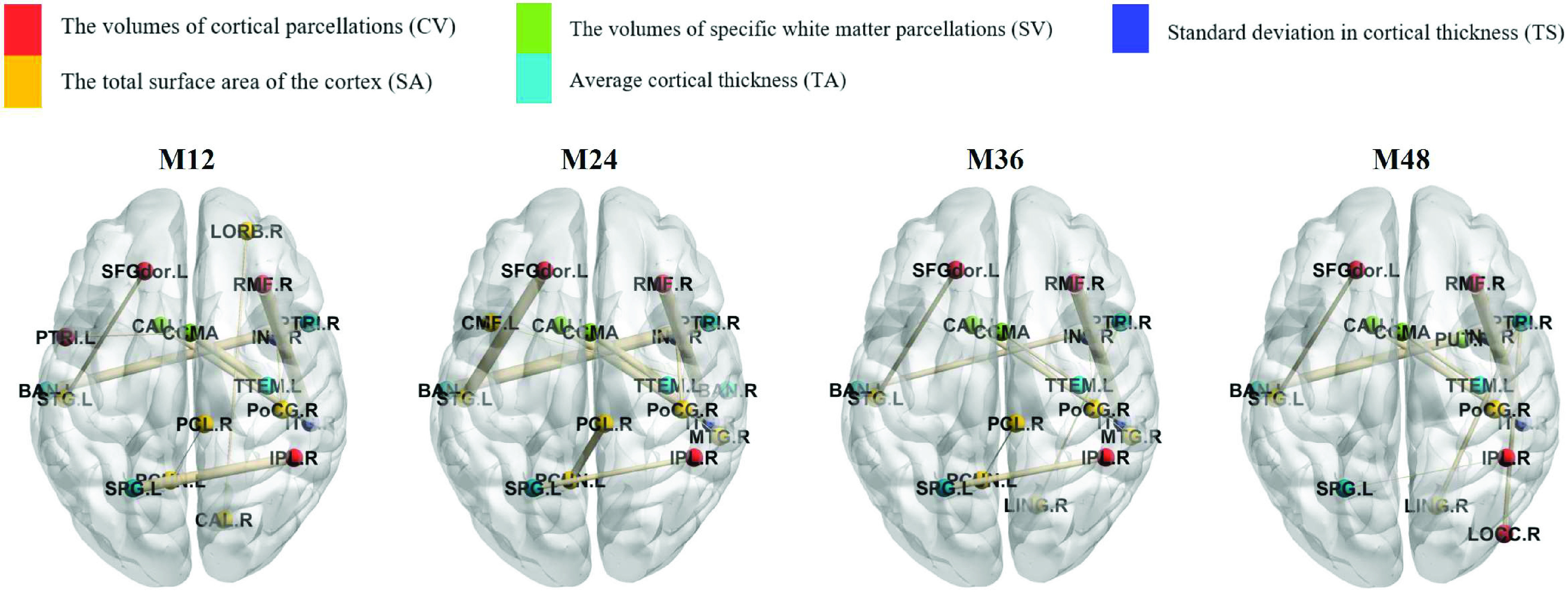
Visualization for the top-10 rank brain biomarker relationships with different time points for the proposed TMTL-GB model on MMSE prediction. Visualization was performed by the toolkit of BrainNet Viewer [40]. The colors of the nodes represent the different biomarker categories and the thickness of the edges represents the importance of the relationship between the biomarkers, with thicker edges representing more important relationships between the biomarkers.

In Fig. 4, we observed a certain similarity in the plots of important brain biomarker relationships at different time points, indicating that there are a number of brain biomarker relationships that are consistently important in the progression of AD and that they can be utilised as potential indicators for the early identification of AD, and in combination with the above tables we discovered that several spatio-temporal relationships between brain biomarkers were significant at all time points. Specifically, they are Vol(C). of R.RostralMiddleFrontal - Surf. Area of R.InferiorTemporal, CTA. of L.SuperiorParietal - Vol(C). of R.InferiorParietal, CTA. of R.ParsTriangularis - CTA. of L.Bankssts, Vol(WM). of CorpusCallosumMidAnterior - CTA. of L.TransverseTemporal, Vol(WM). of L.Caudate - CTStd. of R.InferiorTemporal, Vol(C). of L.SuperiorFrontal - Surf. Area of L.SuperiorTemporal and Surf. Area of R.Postcentral - CTStd. of R.Insula.

For Vol(C). of R.RostralMiddleFrontal - Surf. Area of R.InferiorTemporal, the frontal gyrus is related to a person’s literacy and numeracy skills [41]; The inferior temporal gyrus is closely related to visual information processing, and abnormalities of the inferior temporal gyrus are associated with semantic memory disorders (e.g. AD), face blindness and cortical color blindness [42]. Both are related to the processing and implementation of information in the brain.

For CTA. of L.SuperiorParietal - Vol(C). of R.InferiorParietal, the superior parietal lobule is associated with spatially oriented brain functions; The inferior parietal lobule is associated with emotional cognition and the interpretation of sensory information, as well as with language, mathematical operations and bodily imagery [43]. This correlation can be a factor in causing decline estimation and analytical ability in AD.

For CTA. of R.ParsTriangularis - CTA. of L.Bankssts, the pars triangularis is associated with the ability to translate from a second or third language back into the mother tongue, with its involvement in semantic processing [44]; Bankssts is the posterior part of the superior temporal gyrus, which is responsible for processing auditory signals, including speech, and the comprehension of language [45]. Both are correlated with the brain’s linguistic response and semantic processing.

For Vol(WM). of CorpusCallosumMidAnterior - CTA. of L.TransverseTemporal, the corpus callosum is a fibrous bundle of fibers linking the left and right hemispheres. It maintains coordinated activity between the two hemispheres and connects the corresponding parts of the left and right hemispheres, coordinating the activity between the two halves of the brain and making the brain function as one. If the corpus callosum is impaired, the activity of the two hemispheres is not coordinated [46]; The transverse temporal gyrus is the first cortical structure to process auditory information and is part of the primary auditory cortex [47]. This correlation can be one of the factors causing reduced physical coordination in AD.

For Vol(WM). of L.Caudate - CTStd. of R.InferiorTemporal, the caudate nucleus is an important part of the brain’s learning and memory system [48]; The inferior temporal gyrus is closely related to visual information processing. This correlation can be one of the factors that cause the decline of learning ability and memory in AD.

For Vol(C). of L.SuperiorFrontal - Surf. Area of L.SuperiorTemporal, the superior frontal gyrus is involved in higher order cognitive functions of the brain, particularly working memory [49]; The superior temporal gyrus includes parts of the auditory cortex as well as the main areas of the language center [45]. This correlation can be a factor that causes the co-occurrence of language function and cognitive dysfunction in AD patients.

For Surf. Area of R.Postcentral - CTStd. of R.Insula, the postcentral gyrus is the seat of the primary somatosensory cortex and is the nerve center of the somatosensory system [50]; The insula is thought to be associated with consciousness and to play a role in a variety of functions normally associated with the regulation of emotion or bodily homeostasis, these functions include perception, motor control, self-awareness, cognitive functions [51]. This correlation can be one of the factors that cause physical activity impairment in AD.

AD is clinically characterized by generalized dementia manifestations such as memory impairment, agnosia, aphasia, apraxia, impairment of visuospatial skills, personality and behavioural changes, and executive dysfunction. We can observe that the important spatio-temporal relationships between brain biomarkers indicative of brain functions are all related to the clinical manifestations of AD.

### C. Clinical Application

In clinical application, our proposed approach can be utilised to obtain a patient’s current MRI data and predict the patient’s cognitive scores at multiple time points in the future, thereby helping clinicians and patients to detect the disease and implement intervention treatment in early stages. Moreover, patients suspected of AD will continue to go to the hospital for MRI testing in real-word application. Subsequent incremental MRI data is wasted if only the baseline model is used or if the patient’s serial examination records cannot be properly integrated. To address this problem, we have applied the concept of ensemble learning to our approach, which allows the model to continuously receive MRI data from subjects and continuously update predictions of future cognitive scores with improved accuracy.

## VI. Conclusion

We proposed a tensor multi-task ensemble learning method based on tensor decomposition for predicting AD progression at different time points to overcome variability and instability in prediction accuracy. In our framework, a prediction model is developed based on spatio-temporal morphological variation trend correlations across biomarkers and multi-task regression, utilising tensor latent factors as multi-task relationships to transfer knowledge and calculate final prediction results. Furthermore, the proposed approach utilises gradient boosting ensemble learning technique integrate temporally continuous MRI recordings to consistently improve predictive accuracy of AD progression. The results of the experiment demonstrate that correlation information can be utilised to identify variations in individual brain structures underlying AD, MCI, and CN, and support the utilisation of correlation data to predict and diagnose AD progression.
